# Dental associations with blood mercury in pregnant women

**DOI:** 10.1111/cdoe.12208

**Published:** 2015-12-21

**Authors:** Jean Golding, Colin D. Steer, Steven Gregory, Tony Lowery, Joseph R. Hibbeln, Caroline M. Taylor

**Affiliations:** ^1^ Centre for Child and Adolescent HealthSchool of Social & Community MedicineUniversity of BristolBristolUK; ^2^ National Seafood Inspection LaboratoryNational Marine Fisheries ServiceNational Oceanic & Atmospheric AdministrationPascagoulaMSUSA; ^3^ National Institute on Alcohol Abuse and AlcoholismNational Institutes of HealthBethesdaMDUSA

**Keywords:** ALSPAC, blood mercury, dental amalgam, pregnancy, seafood

## Abstract

**Objectives:**

There is curiosity concerning the source of mercury that is absorbed into the mother's blood and which may affect the developing fetus. This study therefore sets out to determine the extent to which dental amalgam (DA) may contribute to total blood mercury (TBHg) levels of pregnant women in the UK.

**Methods:**

Whole blood samples and information on diet and socio‐demographic factors were collected from pregnant women (*n* = 4484) enrolled in the Avon Longitudinal Study of Parents and Children (ALSPAC). The whole blood samples were assayed for total mercury levels using inductively coupled plasma dynamic reaction cell mass spectrometry (ICP‐DRC‐MS), and the women were retrospectively asked about features of their dental care during the pregnancy. Linear regression was used to estimate the relative contributions of DA to TBHg levels (log‐transformed) based on *R* ^2^ values, compared to the results from dietary and socio‐demographic variables.

**Results:**

The contribution to the variance of the mothers' TBHg levels by dental variables was 6.47%, a figure comparable to the 8.75% shown for seafood consumption in this population. Dietary and dental variables explained 20.16% of the variance, with socio‐demographic variables contributing only a further 3.40%. The number of amalgams in the mouth at the start of pregnancy accounted for most of the variance in dental variables.

**Conclusions:**

Dental amalgam contributes a comparable amount of variance in TBHg to seafood consumption in this population. However, because the measures of DA exposure were imprecise, these findings are likely to be an underestimate. There is no evidence to date in the literature that fetal exposures to mercury from maternal DAs have adverse effects on the developing child, but long‐term studies are warranted.

There has been considerable and ongoing concern as to the adverse consequences of mercury on health and development. Most concern relates to mercury from seafood, but we have shown, using a population of pregnant women, that seafood contributes a relatively small amount of the variance of total blood mercury (TBHg) levels^1^. The question raised at the end of our publication concerned the origin of the other environmental contributors to blood mercury. In this article, we assess the relative contribution of the mother's dental amalgam (DA) in comparison with dietary influences using the same dataset.

Although it is tempting to assume that the mercury in DA is inert, there is now evidence in humans that mercury vapor is released continuously from amalgams, and that this results in a marked increase during chewing^2^. Given this, it is unsurprising that the amount of mercury in exhaled breath is correlated with the number of DA fillings in the mouth^3^, and that this number is also related to the concentration of mercury in various tissues in the body, particularly the brain^4^. That the mercury in these tissues derives from the amalgam has been shown experimentally using mercury radioisotopes^2^, ^5^.

Mercury from DA is found in the urine^6^, has been shown to cross the placenta^7^, and is found in amniotic fluid, cord blood, and fetal tissues including the brain^2^, ^3^. That the level of mercury in the fetus is strongly correlated with the number of DA fillings has been illustrated by study of cord blood^8^. However, a small postmortem study of 20 second trimester fetuses showed an increasing accumulation of mercury in the fetal kidney (but not the brain) with increasing number of amalgams^9^. Assuming that the lack of association with the brain was not a function of small numbers, it may indicate that accumulation of mercury in the fetal brain only occurs in the third trimester.

There has been some controversy concerning whether DA would have adverse effects as inorganic as opposed to methyl mercury is assumed to be relatively inert. However, Leistevuo^10^ examined the saliva of 187 adults and showed that both the inorganic and organic mercury (mostly methylmercury) levels were correlated with the number of amalgams (*r* = 0.46 and 0.27, respectively). The authors concluded ‘our results are compatible with the hypothesis that amalgam fillings may be a continuous source of organic mercury, which is more toxic than inorganic mercury and almost completely absorbed by the human intestine’. At the same time, Vahter^11^ published a study of 148 pregnant women and reported that 72% of the mercury in their blood was methylmercury. Thus, we can assume that total methylmercury levels in blood will include mercury derived from DAs as well as from dietary components such as seafood.

In our earlier publication^1^, we showed that 19.8% of the variance in the natural log (ln) maternal TBHg was provided by 103 features of the diet. Less than half of this variance was provided by seafood. There were also independent socio‐demographic features (the better the social circumstances the higher the level) that contributed to the variance of ln TBHg. In this article, we use the same cohort to assess the extent of the contribution of DA to the mothers' TBHg level and determine whether dental care may explain some of the socio‐demographic variation.

## Materials and methods

### The ALSPAC study

All pregnant women resident in a geographic area (Avon) in the UK, whose expected date of delivery lay between the 1 April 1991 and 31 December 1992, were eligible to take part in the Avon Longitudinal Study of Parents and Children (ALSPAC). A total of 14 541 pregnancies were enrolled: over 80% of the eligible pregnancies^12^. The aims of the study were to determine ways in which the individual's genotype combines with environmental pressures (particularly those operating prenatally and/or in infancy) to influence health and development^13^, ^14^. Data were obtained using a variety of methodologies: relevant to this study were self‐completion questionnaires completed by each parent, and assays of biological samples, as described below. Women were offered the assistance of an interpreter or interviewer if they did not speak English or needed help to complete the questionnaire. The study website contains details of all the data that are available through a fully searchable data dictionary: http://www.bris.ac.uk/alspac/researchers/data-access/data-dictionary/.

Ethical approval for the study was obtained from the ALSPAC Law and Ethics Committee and the Local Research Ethics Committees. (Consent for questionnaire completion was implied if the questionnaire was completed and returned to the study office – there was no compulsion to do so, and no reward was given; analyses of biological samples were only carried out with written permission).

### Collection of blood samples for trace metal estimations

Whole blood samples were collected in acid washed heparin vacutainers (Becton and Dickinson) by midwives as early as possible in pregnancy. This was purely voluntary on their part as the study had no funding for this. Samples were obtained from women in two of the three Health Authority areas of the recruitment region. Altogether, there were 4484 samples collected. The women who gave such samples were slightly older and slightly better educated than the rest of the cohort^15^. Samples were stored at 4°C at the collection site and then sent to the central Bristol laboratory within 0–4 days. These samples were at room temperature for up to 3 h during transfer, and were stored at 4°C as whole blood in the original tubes for 18–19 years before being sent for analysis. Gestations when blood samples were taken were available for 4472 mothers (99.7%); 93% of gestations were <18 weeks with interquartile range 9–13 weeks.

### Analysis of samples

The Centers for Disease Control and Prevention (CDC) developed methods to prepare the samples for analysis of whole blood mercury as well as of lead, selenium, and cadmium (CDC method 3009.1) as described in our previous publication^1^. In brief, clotted whole blood was digested to remove all clots, before being analyzed using inductively coupled plasma dynamic reaction cell mass spectrometry (ICP‐DRC‐MS). Two levels of bench quality control (QC) materials as well as a blind QC material were used for daily QC. Of the 4484 samples, the assay failed for 353, and 4131 had valid results for mercury with three of these having readings that were below the limit of detection (LOD) of the assay (0.24 μg/l). For these samples, a value of 0.7 times the LOD value was ascribed as this was seen to be most likely from the overall distribution of the mercury levels.

### The dental measures

The questions concerning dental treatment of the mother were not asked until 2 years after the study child was born. At this point, the mother was asked the following: (i) The number of fillings she had in her mouth at the time she became pregnant (none; 1; 2–3; 4 or more); (ii) during the study pregnancy whether she had visited the dentist at all (yes/no); (iii) if yes to (ii), (a) did she have any teeth out? (yes/no); (b) did she have new amalgam fillings put in? (yes/no); (c) did she have old amalgam fillings removed? (yes/no); (d) did she have dental gas? (yes/no); (e) did she have a dental X‐ray (yes/no)? For questions (a) – (e) those who had not visited the dentist were coded ‘no’. These questions were answered by 90% or more of the women answering this questionnaire – the question with lowest response was that relating to the number of amalgams at the start of pregnancy, where 10% stated that they could not remember. It is important to note that pregnant women were provided with free dental treatment as part of the UK's National Health Service, in consequence of which many women took advantage of this and obtained treatment they otherwise would be likely to have postponed. Thus, there was unlikely to be any reduction in dental care in pregnancy due to lack of finance.

### The dietary measures

An assessment of prenatal dietary consumption used a food frequency format, asking the number of occasions per time interval the woman currently ate specific types of food, and additionally the most frequently used milk, fats, and types of bread consumed^16^. These questions were validated by showing strong correlations between the frequency of oily fish consumption with prenatal blood levels of the omega‐3 fatty acid docosahexaenoic acid (DHA)^17^.

### Socio‐demographic factors

The following socio‐demographic factors were collected using questionnaires completed by the mother: (i) her age at the time of the delivery of the child; (ii) her social class based on her last/current occupation, graded from I (professional) to IV (semi‐skilled) and V (unskilled manual workers); (iii) her highest education level achieved, using 5 ranked categories from minimal to university degree; (iv) housing tenure, distinguishing between mortgaged or owned outright, rented public housing, and other accommodation; (v) her ethnic background distinguishing white and non‐white; and (vi) her parity, defined as the number of previous pregnancies resulting in a live or stillbirth.

### Statistical analyses

As the distribution of mercury levels was slightly skewed, we employed a natural log (ln) transformation for all analyses. To develop a final model for the dental variables, we offered them all to a stepwise multiple regression with *P* value to enter of 0.05, and eliminated those variables that did not enter.

An estimate of the contribution of DA to TBHg levels was based upon the *R* ^2^ for simple linear regression models involving the dental variables available. To assess whether the dental components were a consequence of socio‐demographic or dietary influences, or *vice versa,* the *R* ^2^ attributed to each, and combinations of the three were computed.

## Results

Details of maternal dental care relevant to the current research question were available for between 2591 and 2855 women for whom prenatal TBHg levels were available. As shown in Table 1, there were few differences between the dental care of women with and without mercury levels available.

**Table 1:** Positive responses [% (*n*)] to questions on dental amalgam (DA) fillings at 33 months comparing those with and without blood mercury level

	Mercury measure available	No mercury measure
4+ DAs at start of pregnancy	69.6% (1823)	69.9% (4200)
Visited dentist in pregnancy	86.3% (2464)	86.0% (5765)
Had a new DA in pregnancy	22.4% (633)	25.0% (1632)
Had old DA removed in pregnancy	14.8% (420)	15.7% (1021)
Tooth extraction in pregnancy	3.8% (108)	3.9% (251)

As anticipated, there were the expected unadjusted differences between the arithmetic mean TBHg levels and features of dental care (Table 2). Women who had visited the dentist, had old amalgams extracted, or new ones inserted had significantly greater levels of mercury. Although those who had at least 4 DAs in place had markedly higher levels of TBHg, there was little difference between the levels of the women who had zero and 1–3 amalgam fillings. These two groups have therefore been combined for further analyses. In contrast the women who had had dental extractions had reduced mercury levels.

**Table 2:** Relationship between the unadjusted mean total blood mercury level in pregnancy and features of dental care as ascertained at 33 months

Features of dental care	*n*	Mean (SD) total blood Hg μg/l	*P*
No. of dental amalgams (DAs) at start of pregnancy			
None	207	1.84 (1.04)	<0.0001
1–3	586	1.90 (1.16)	
4+	1798	2.28 (1.05)	
Visited dentist during pregnancy			
Yes	2464	2.20 (1.09)	<0.0001
No	391	1.88 (1.11)	
Had new DAs in pregnancy			
Yes	625	2.34 (1.18)	<0.0001
No	2171	2.11 (1.06)	
Had old DAs removed in pregnancy			
Yes	413	2.35 (1.02)	<0.0001
No	2383	2.13 (1.11)	
Tooth extraction in pregnancy			
Yes	107	1.99 (1.13)	0.099
No	2689	2.17 (1.09)	

The next step was to assess the independent relationships between the variables in Table 2 and maternal ln TBHg levels. The predominant variable concerned the presence of at least 4 amalgam fillings in the mouth at the start of pregnancy (Table 3). Visiting the dentist was associated with higher levels of blood mercury, independent of treatment such as having a new filling, although it may have been a partial proxy for the effects of removal of fillings and the negative effect of tooth extraction. The overall *R* ^2^ for these four dental variables remaining in the model was 6.47%. Although the final model reported in Table 3 was the best in terms of explanation, an alternative model performed similarly. For example, substituting fillings removed for new fillings inserted achieved *R* ^2^ = 6.46%; this is not surprising as removal of a filling usually results in insertion of another.

**Table 3:** Linear regression of log mercury on dental variables after stepwise reduction (*n* = 2572, *R* ^2^ = 6.47%)

Features of dental care in pregnancy	b (95% CI)	*P*
No. DAs in pregnancy 4+ versus <4	0.198 (0.159, 0.238)	1.1 × 10^−22^
Visited dentist in pregnancy	0.161 (0.105, 0.217)	2.1 × 10^−8^
New DA in pregnancy	0.053 (0.008, 0.097)	3.8 × 10^−4^
Tooth extracted in pregnancy	−0.174 (−0.271, −0.078)	0.0195

b: regression coefficient (μg/l); DAs, dental amalgams.

### Sensitivity analyses

To assess the impact of recall bias, the responses to visiting the dentist and new fillings at 33 months were compared to a subgroup of the cohort who was asked these questions at 2 months postdelivery. Agreements were 88% and 79%, respectively (*n* = 1690, *P* < 0.001). As a sensitivity analysis, we used the information collected at 2 months post‐delivery concerning whether the mother had visited a dentist during pregnancy. The regression model was similar to that shown with the 33‐month measure, but the proportion of variance explained (*R* ^2^) had resulted in an increase to 7.67%, thus indicating an improvement in accuracy. However, this measure was only available for 1533 subjects.

### Comparison of dental, social, and dietary relationships

In Table 4, we compare the various combinations of the groups of dental, social, and dietary variables. If the variables were mutually distinct, the total *R* ^2^ would be as high as 36.13% (Dental alone + Socio‐demographic alone + Diet alone). However, the actual value of *R* ^2^ on combining all these variables into one model is 23.56%, implying that many of the variables are mutually associated. If we concentrate on the two types of variables that are shown in the literature to be associated with blood mercury (dental and dietary), the *R* ^2^ rises to 20.16%. Although on their own the socio‐demographic variables account for an *R* ^2^ of 12.61% (B), addition of these variables to the model containing dental and dietary variables (G) results in an increase of only 3.40% (23.56–20.16%). This implies that the socio‐demographic variables, which we have shown elsewhere to indicate positive associations (more advantageous living conditions) with TBHg^1^, largely operate through dietary preferences and use of dental care. Further analyses at the individual variable level showed that the individual social variables maternal education and housing tenure were no longer associated in the presence of dental and dietary variables. However, the demographic markers maternal age and parity were still strongly and independently associated and are likely to have biological explanations.

**Table 4:** Comparisons of variances attributable to dietary, socio‐demographic, and dental factors to the mother's total blood mercury

Group of factors	*R* ^2^ (%)
A. Dental alone	6.47
B. Socio‐demographic alone	12.61
C. Dietary alone	17.05^a^
D. Dental + Socio‐demographic	14.83
E. Dental + Dietary	20.16
F. Socio‐demographic + Dietary	21.38
G. Dental + Socio‐demographic + Dietary	23.56

*n* = 2346.

a Difference from published figure of 19.8% in reference 1 due to a change in sample size in the current analyses.

## Discussion

In this study, we have used a relatively large population of pregnant women to demonstrate that dental care, particularly the number of DA fillings, is responsible for at least 6.47% of their TBHg level. This may be compared with the 8.75% we have shown to be attributable to seafood consumption in the same population^1^. Clearly finer detail of the subjects' dental history (such as the actual number of amalgam fillings inserted) would have been ideal and would be likely to have been associated with an increase in the amount of variance explained. The importance of accuracy was demonstrated when the dental history collected at 2 months postdelivery was compared with that collected at 2 years, which was associated with an increase in variance from 6.47% to 7.67%.

Importantly, this study is concerned with population levels of TBHg and the factors that influence them. We have shown that it is likely that, in this population, the variation in maternal blood mercury levels attributable to seafood is similar to that attributable to dental care using DA.

In accord with our study, there have been reports of positive associations between blood mercury and age^18^, ^19^, and maternal blood mercury levels have been associated with higher education, and income^20^, ^21^. In the UK, it is true that women with higher levels of education and/or income tend to eat fish more frequently; it is also true that women in these social groups tend to be more assiduous in obtaining dental treatment even though such treatment is free for pregnant women. Thus, it is not surprising that these socioeconomic associations with blood mercury can be explained by a combination of diet and DA treatment.

The present study has a number of advantages and disadvantages. The disadvantages were (i) that the questions on DA were asked about 2 years after the end of pregnancy; consequently, the memory of the participating mothers is likely to be faulty. It is probable, however, that women who had ≤3 fillings would be able to remember and report this, but those with larger numbers of fillings would be less likely to recall the exact number. This was found in a validation study of pregnant women in Norway where the women were asked to use a mirror to count the number of amalgams in their mouths; the correlation with a dental examination was high for those with <5 fillings but much poorer for those with higher numbers^22^. For this reason, we deliberately designed the questionnaire to ask the woman to estimate whether 0, 1, 2–3, or 4 or more fillings were in place. (ii) Other consequences of recall bias were addressed by comparing results from a subgroup of women who gave the answers at 2 months postdelivery with their responses 31 months later (see Sensitivity analyses). We showed that the variance explained was greater with the earlier measure, thus implying that earlier ascertainment would have resulted in improved accuracy. (iii) We were unable to determine whether the women who had had dental treatment in pregnancy had done so before or after the blood was drawn – the consequence is that the relationship between blood mercury and dental care is likely to be an underestimate. (iv) The whole blood was stored at 4°C for 19 years, and consequently posed problems for analysis^1^ – it has been suggested that some of the mercury would be likely to be absorbed into the glass. However, any differences are likely to be consistent across the samples and are unlikely to obviate the relationships we have shown.

Among the advantages are that compared with most other studies: (i) the numbers are large, (ii) the participants were population based, (iii) questions on DA were inserted in the middle of the questionnaire without reference to any outcome, and thus, there was little chance of the study subject answering the questions in a biased way.

There has been confusion as to how safe DA is for the population in general; for example, in 2006 Bates^23^ reviewed the evidence in regard to adverse effects of DA fillings on (mainly adult) health and disease and showed that there were few sound epidemiological studies. More reliable studies have been undertaken to assess whether maternal DA has any adverse effects on the developing brain of the fetus. This has been tested in two birth cohort studies. (i) In the Seychelles, a detailed study showed no effect of the number of amalgams in pregnancy and child motor development at 9 and 30 months^24^ or neurodevelopment at age 5 years^25^; there were no adverse associations with educational achievement, language or other cognitive outcomes at 5 years old^26^. (ii) An analysis of the ALSPAC cohort demonstrated no relationship between speech and language development at 15 months and any measure of prenatal DA exposure^27^. To our knowledge there have been no studies to date following exposed children beyond the age of 5 years. More studies are needed to determine whether there are long‐term effects.

A phase down of the use of mercury was agreed at the Minamata Convention in 2013^28^. However, amalgam is still widely used in the UK^29^ and cessation of its use will take several years to complete: there needs to be considerable changes in dental school teaching and continuing professional development curricula to bring about updated clinical practice^30^. Mercury exposure from DA is therefore potentially an ongoing problem, in the UK at least. Although we have indicated that as much as 20% of the variance in TBHg may be contributed by a combination of dietary and dental features, it is well known that mercury can also be absorbed from water and air. Mercury vapor in the atmosphere is absorbed mainly through the respiratory tract^31^. Once absorbed the mercury is widely distributed to fat‐rich tissues and is readily transferred across the placenta and blood–brain barriers. It has been estimated that 9.9 tons of mercury are deposited on the UK from the atmosphere each year (41% from sources in the UK, 33% from elsewhere in Europe, and 25% from other parts of the northern hemisphere)^32^. It is very likely that such sources contribute a considerable proportion of TBHg, but is impossible to assess accurately in this study. Even though our measures of prenatal amalgam exposure are less than perfect, we have shown that there is a detectable contribution to the maternal TBHg level.

## Competing financial interests

The authors have no competing interests.
